# La luxation acromio claviculaire stade III du sujet âgé

**DOI:** 10.11604/pamj.2015.21.225.7448

**Published:** 2015-07-30

**Authors:** Samia Frioui, Sonia Jemni

**Affiliations:** 1 Service de Médecine Physique et de Réadaptation Fonctionnelle, CHU Sahloul, Faculté de Médecine “Ibn El Jazzar”, Sousse, Tunisie

**Keywords:** Luxation acromio-claviculaire, épaule, Stade III, sujet âgé, acromioclavicular dislocation, shoulder, Stade III, elderly

## Image en medicine

Les luxations acromio-claviculaires constituent une préoccupation des médecins et des chirurgiens depuis très longtemps puisque Hippocrate en fournissait déjà une description précise. Elles représentent 8% des traumatismes de la ceinture scapulaire, touchant préférentiellement une population masculine et rencontrée régulièrement en pratique sportive. Si le traitement des stades I et II est fonctionnel, le traitement chirurgical est largement proposé pour les stades IV et au-delà. Le traitement du stade III reste un sujet de controverse entre les tenants du traitement conservateur et les partisans du traitement chirurgical. Entre les années 1940-1960, le traitement chirurgical était de mise pour toutes les pathologies acromio-claviculaires, quel que soit le stade. Nous rapportons le cas d'un patient âgé de 73 ans, hypertendu, droitier, qui suite à une chute de sa propre hauteur avec réception sur l’épaule gauche a présenté une douleur avec impotence fonctionnelle du membre supérieur gauche. L'examen de l’épaule objectivait un aspect en coup de hache externe, la clavicule remontait en « touche de piano », on notait une limitation douloureuse des amplitudes articulaires de l’épaule avec une douleur élective à la palpation de l'articulation acromio-claviculaire. Le bilan radiologique confirmait le diagnostic de luxation acromio-claviculaire stade III. Une indication chirurgicale a été posée mais refusée par le patient. Il a alors bénéficié de séances de rééducation avec une nette amélioration des douleurs. Il a récupéré une mobilité fonctionnelle et a été satisfait du résultat. Actuellement, les traitements conservateurs ont largement remplacé les indications opératoires et les résultats subjectifs et objectifs sont meilleurs.

**Figure 1:**
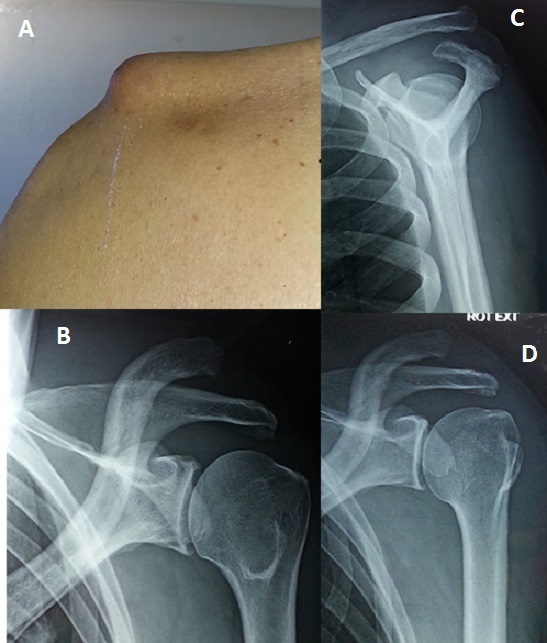
(A) épaule vue du dos: on note que la clavicule remonte en « touche de piano »; (B) radiographie de l’épaule de face: la disjonction acromio-claviculaire est nettement visible; (C) radiographie de l’épaule de profil: disjonction acromio-claviculaire; (D) radiographie de l’épaule en rotation externe montrant la disjonction acromio-claviculaire

